# Identification of LRP-1 as an endocytosis and recycling receptor for β1-integrin in thyroid cancer cells

**DOI:** 10.18632/oncotarget.20201

**Published:** 2017-08-10

**Authors:** Louis Theret, Albin Jeanne, Benoit Langlois, Cathy Hachet, Marion David, Michel Khrestchatisky, Jérôme Devy, Emonard Hervé, Sébastien Almagro, Stéphane Dedieu

**Affiliations:** ^1^ Université de Reims Champagne-Ardenne, UFR Sciences Exactes et Naturelles, Reims, France; ^2^ CNRS UMR 7369, Matrice Extracellulaire et Dynamique Cellulaire, MEDyC, Reims, France; ^3^ SATT Nord, Lille, France; ^4^ VECT-HORUS SAS, Faculté de Médecine Secteur Nord, Marseille, France; ^5^ Aix Marseille Université, CNRS, NICN, Marseille, France

**Keywords:** LRP-1, β1-integrin, cancer, endocytosis, recycling

## Abstract

LRP-1 is a large endocytic receptor mediating the clearance of various molecules from the extracellular matrix. LRP-1 was reported to control focal adhesion turnover to optimize the adhesion-deadhesion balance to support invasion. To better understand how LRP-1 coordinates cell-extracellular matrix interface, we explored its ability to regulate cell surface integrins in thyroid carcinomas. Using an antibody approach, we demonstrated that β1-integrin levels were increased at the plasma membrane under *LRP1* silencing or upon RAP treatment, used as LRP-1 antagonist. Our data revealed that LRP-1 binds with both inactive and active β1-integrin conformations and identified the extracellular ligand-binding domains II or IV of LRP-1 as sufficient to bind β1-integrin. Using a recombinant β1-integrin, we demonstrated that LRP-1 acts as a regulator of β1-integrin intracellular traffic. Moreover, RAP or LRP-1 blocking antibodies decreased up to 36% the number of β1-integrin-containing endosomes. LRP-1 blockade did not significantly affect the levels of β1-integrin-containing lysosomes while decreasing localization of β1-integrin within Rab-11 positive vesicles. Overall, we identified an original molecular process in which LRP-1 acts as a main regulator of β1-integrin internalization and recycling in thyroid cancer cells.

## INTRODUCTION

The low-density lipoprotein receptor-related protein-1 (LRP-1) is a large multifunctional endocytic receptor belonging to the low-density lipoprotein receptor family. First described for its role in lipoprotein metabolism [1], LRP-1 has since been shown to be involved in many physiological and pathological processes including control of the hepatic function and cholesterol homeostasis [2], vascular integrity [3] and blood-brain barrier permeability [4] as well as Alzheimer disease [5] and cancer development [6]. While initially synthesized as a 600 kDa precursor, LRP-1 is rapidly cleaved by a furine convertase into two non-covalently linked chains, i.e. a 515 kDa α-chain and an 85 kDa transmembrane β-chain comprising the intra-cytoplasmic domain. The α-chain is exclusively extracellular and holds four cystein-rich domains allowing LRP-1 to interact with more than thirty soluble or membrane-anchored ligands. The intracytoplasmic domain of the β-chain consists of a short 100 amino-acid cytoplasmic tail containing two NPXY motifs with the distal one overlapping an YXXL sequence, which are crucial for recruiting molecular adaptors as well as signaling proteins and to trigger endocytosis, respectively [7].

Through its endocytic activity, LRP-1 mediates internalization and catabolism of various extracellular proteinases, therefore limiting extracellular matrix (ECM) remodeling [8, 9]. Clearance of the uPA (urokinase plaminogen activator receptor)/PAI-1 (plasminogen activator inhibitor-1) complex can be achieved through uPAR binding to LRP-1, thus leading to uPA:PAI-1 lysosomal degradation, uPAR recycling and subsequent regulation of the plasmin activation cascade [10]. In addition, LRP-1 has been shown to be a key player in the clearance of matrix metalloproteinases (MMPs) i.e. MMP-2 [11], -9 [12], -13 [13] but also ADAMTS (a disintegrin and metalloproteinase with thrombospondin motifs)-4 [14] and ADAMTS-5 [15]. The aspartic protease cathepsin-D, whose overexpression is correlated with poor prognosis in breast cancer, is also endocytosed by LRP-1 [16]. As a consequence of its well-identified function in controlling extracellular proteolysis, LRP-1 was initially considered as preventing tumor aggressiveness [8]. This was reinforced by studies conducted in rodents or considering human samples that both further confirmed the existing correlation between low LRP-1 expression levels and poor survival [17–19]. Despite these experimental and clinical data, the overall contribution of LRP-1 to tumor progression appears actually much more complex, remaining mostly misunderstood and somehow controversial. Previous work has reported LRP-1 as supporting invasion, survival or metastatic dissemination of thyroid carcinoma [20, 21] and breast cancer cells [22–24]. Consistently, C766T *LRP1* gene polymorphism, which has been previously associated with neurodegenerative disease [25], also correlates with increased breast cancer occurrence [26]. More recently, an elegant network-based exploratory study found LRP-1 as being highly connected to a multi-cancer gene expression biomarker, which appears to be strongly predictive of clinical outcome in 12 types of cancers [27]. In order to comprehensively clarify the function of this endocytic receptor in tumor cells, others have sought to decipher LRP-1-related molecular mechanisms and signaling pathways. Since then, it has been demonstrated that the cell surface expression of LRP-1 is frequently increased at the invasive front, especially within adhesion and actin-rich structures [22, 28]. In addition, LRP-1 interaction with membrane-bound calreticulin was shown to stimulate focal adhesion reorganization in non-cancer cells [29]. In a tumor context, we previously demonstrated that LRP-1 controls actin cytoskeleton organization and focal adhesion complex turnover [20, 30]. LRP-1 is required to ensure the appropriate distribution of paxillin and FAK (focal adhesion kinase) within focal adhesions and contributes to optimize thyroid carcinoma cell adhesion and invasion by supporting ERK (extracellular signal-regulated kinases) and concomitantly inhibiting JNK (c-jun N-terminal kinase) pathways [21]. Furthermore, we recently identified LRP-1 as a main endocytic receptor for the hyaluronan receptor CD44, hence fundamentally regulating tumor cell morphology and ECM attachment [28].

In view of the above, one should consider LRP-1 as a main regulator of cell-matrix interaction dynamics acting *via* coordination of the adhesion-deadhesion balance, especially within a tumor microenvironment. Nevertheless, the relatively poor knowledge of LRP-1 transmembrane interactome impedes our thorough understanding of the way it controls cell-matrix interaction dynamics and contributes to malignant disease progression. Among the range of possibilities, both integrins and LRP-1 appear to be engaged in similar molecular pathways regulating cell adhesion, spreading and motility [31, 32]. With the purpose of establishing an integrated functional relationship between LRP-1-mediated endocytosis and cell-ECM interface, we here explored the ability of LRP-1 to bind cell surface integrins to regulate their uptake and recycling in tumor cells.

## RESULTS

### Cell surface β1-integrin accumulates under LRP-1 inhibition

**Figure 1:**
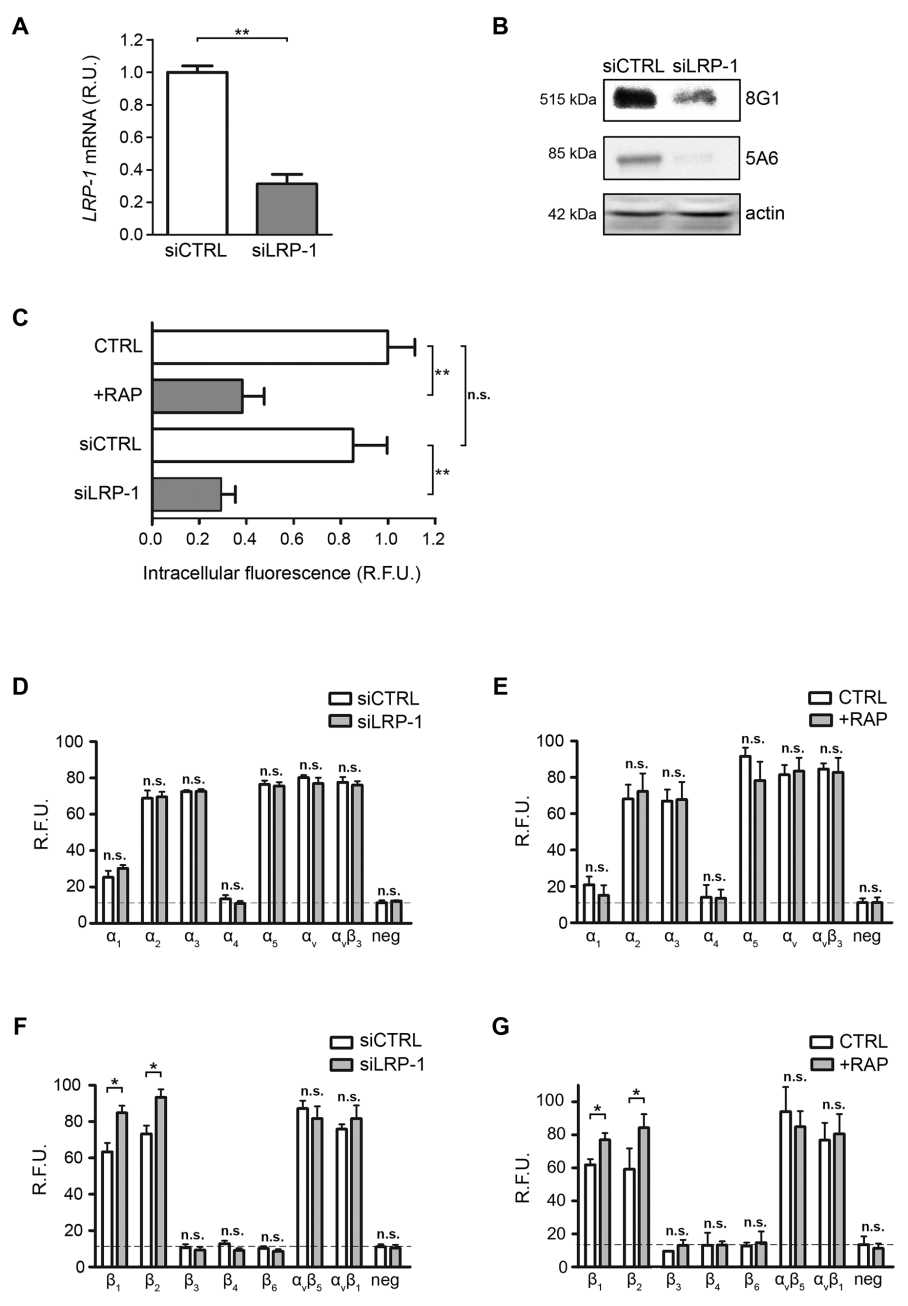
Cell surface β1-integrin accumulates under LRP-1 inhibition or silencing **(A)** Total RNAs were purified from FTC-133 cells transfected with non-silencing siRNA (siCTRL) or siRNA targeting *LRP1* (siLRP-1). *LRP1* mRNA expression was quantified using real-time RT-PCR and expressed as relative units (R.U.). *RPL32* and *RS18* were used for normalization. siCTRL cells served as a reference set to 1. **(B)** LRP-1 protein expression from FTC-133 cells transfected with non-silencing siRNA (siCTRL) or siRNA targeting *LRP1* (siLRP-1) was assessed by SDS-PAGE immunoblotting of LRP-1 α chain (8G1) and LRP-1 β chain (5A6). **(C)** Wild-type FTC-133 cells (WT) were treated in presence or absence of 500 nM RAP or transfected with siRNA sequences (siCTRL and siLRP-1), and then incubated for 30 min in serum-free medium containing FITC-labeled human α2-macroglobulin. The intracellular fluorescence reflecting endocytosis activity was determined as described elsewhere [20] and is expressed as relative fluorescence units (R.F.U.), by comparison with signal from untreated WT cells (CTRL). **(D-G)** α- and β-integrin subunits were quantified at FTC-133 cell surface using an antibody array under *LRP1* silencing (siLRP-1) (D and F) or 500 nM RAP treatment (E and G). The specific signal threshold was established using negative controls (neg) and is represented by dotted lines. Each value is the mean ± SD for at least three independent experiments, each performed in triplicate. n.s., not significant; *, P < 0.05; **, P < 0.01; as compared to the corresponding CTRL or siCTRL condition (white bars).

To assess whether LRP-1 may regulate cell surface integrins, we used both *LRP1* silencing strategy and treatment with the LRP-1 antagonist RAP (receptor-associated protein) in order to inhibit LRP-1-mediated endocytosis. Assays were carried out in FTC-133 cells that remain a favored cellular model of LRP-1 study in the tumor context [20, 28, 33, 34]. Selective *LRP1* silencing was conducted using previously validated short interfering sequences [20] and reached about 70% downregulation of endogenous LRP-1 expression at both mRNA and protein levels (Figure 1A and 1B). LRP-1 ability to mediate endocytosis was then analyzed under these experimental conditions using FITC-labelled α2-macroglobulin as a control ligand [28]. The results confirm that both RAP treatment and LRP-1 silencing inhibit the internalization of labeled substrate by approximately 2- and 3-fold, respectively (Figure 1C). To investigate whether LRP-1 may regulate the level of integrin at the plasma membrane, we then used an antibody array approach to quantify cell surface α- and β-integrin subunits (Figure 1D to 1G). Under control conditions, a wide range of integrins was expressed at the cell surface of FTC-133 thyroid carcinomas, particularly integrin subunits α2, α3, α5, αv, β1 and β2 (Figure 1E and 1G). Neither *LRP1* silencing (Figure 1D) nor its functional inhibition using RAP treatment (Figure 1E) affected the α-integrin subunits expression at the cell surface. Interestingly, both β1 and β2 integrins were found to significantly accumulate at the plasma membrane of FTC-133 cells under *LRP1* silencing, with about 25% increase in measured signals (Figure 1F). None of the other β-integrin subunits appeared affected by *LRP1* downregulation. Consistently, similar results were obtained under RAP treatments (Figure 1G).

### LRP-1 and β1-integrin coexist within the same molecular complexes

**Figure 2:**
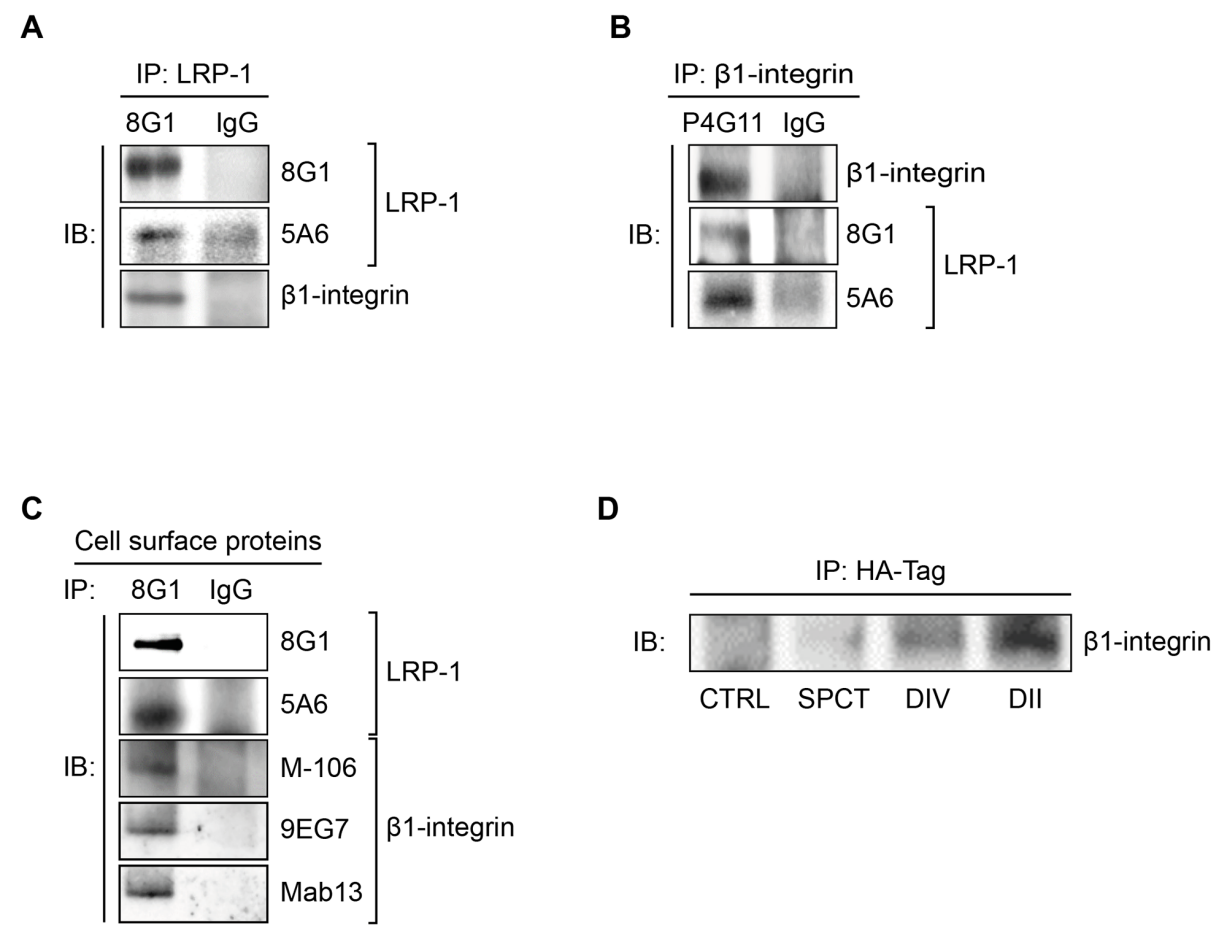
LRP-1 and β1-integrin are found in the same molecular complexes in thyroid cancer cells **(A)** Whole-cell extracts were isolated from FTC-133 cells. Immunoprecipitations of LRP-1 α-chain-containing complexes were carried out using anti-LRP-1 α-chain (8G1), and then isolated immunocomplexes were immunoblotted using anti-LRP-1 α-chain (8G1), anti-LRP-1 β-chain (5A6) and anti-β1-integrin (M-106) antibodies. **(B)** A reverse immunoprecipitation assay was conducted from FTC-133 whole-cell extracts using anti-β1-integrin antibody (P4G11) under experimental conditions described above. **(C)** Biotinylation of FTC-133 cell surface proteins was conducted at 4°C, and biotinylated proteins were isolated using avidin-agarose beads. Cell surface LRP-1-containing complexes were immunoprecipitated using anti-LRP-1 α-chain (8G1) and immunoblotted using 8G1, anti-LRP-1 β-chain (5A6), anti-β1-integrin (M-106), anti-active β1-integrin (9EG7) and anti-inactive β1-integrin (Mab13) antibodies. **(D)** FTC-133 cells were transfected with HA-tagged LRP-1 mini-receptors (SPCT, DIV and DII) or an empty plasmid (CTRL). Immunoprecipitations of HA-tagged LRP-1 mini-receptors were performed using anti-HA antibody (HA-tag) and immunocomplexes were immunoblotted using anti-β1-integrin antibody (M-106). All experiments were repeated three times with different sets of samples and nonspecific IgGs were used as a negative control for immunoprecipitation.

Considering the results displayed in Figure 1F and 1G and that β2-integrin is especially relevant as a leukocyte receptor [35], we investigated whether LRP-1 may interface with β1-integrin. First, co-immunoprecipitation assays were carried out from whole cell extracts (Figure 2A and 2B) and plasma membrane extracts (Figure 2C). The 8G1 antibody, which is directed against the extracellular LRP-1 α-chain, allowed immunoprecipitation of both LRP-1 α- and β-chains (Figure 2A and 2C). Total β1-integrin (M-106) was found co-immunoprecipitated with LRP-1 from whole cell extracts (Figure 2A). Reverse immunoprecipitation experiments with anti-β1-integrin were also performed with the same cell lysates. The data presented in Figure 2B confirmed that LRP-1 and β1-integrin were detected in the same molecular complex. Immunoprecipitations were also conducted using biotinylated proteins to examine cell surface LRP-1-containing complexes. Results displayed on Figure 2C revealed that β1-integrin (M-106) was indeed co-immunoprecipitated with cell surface LRP-1. Using monoclonal antibodies designed to discriminate between active (9EG7) and inactive (Mab13) β1-integrin conformation [36], we further observed that both β1-integrin conformations may bind with cell surface LRP-1 (Figure 2C). Next, in order to determine which part of LRP-1 is involved in β1-integrin binding at the cell surface we overexpressed functional HA-tagged mini-receptors derived from full-length LRP-1 [28]. Immunoprecipitation was conducted from biotinylated cell surface proteins using anti-HA tag (Figure 2D). In this assay, endogenous β1-integrin did not co-immunoprecipitate with the recombinant variant of LRP-1 exhibiting only the LRP-1 β-chain (SPCT). Conversely, LRP-1 constructs harboring the extracellular ligand-binding domains II or IV from the α-chain were sufficient to bind β1-integrin. Of note, larger amounts of endogenous β1-integrin were immunoprecipitated when using LRP-1 ligand-binding domain II-harboring constructs.

**Figure 3:**
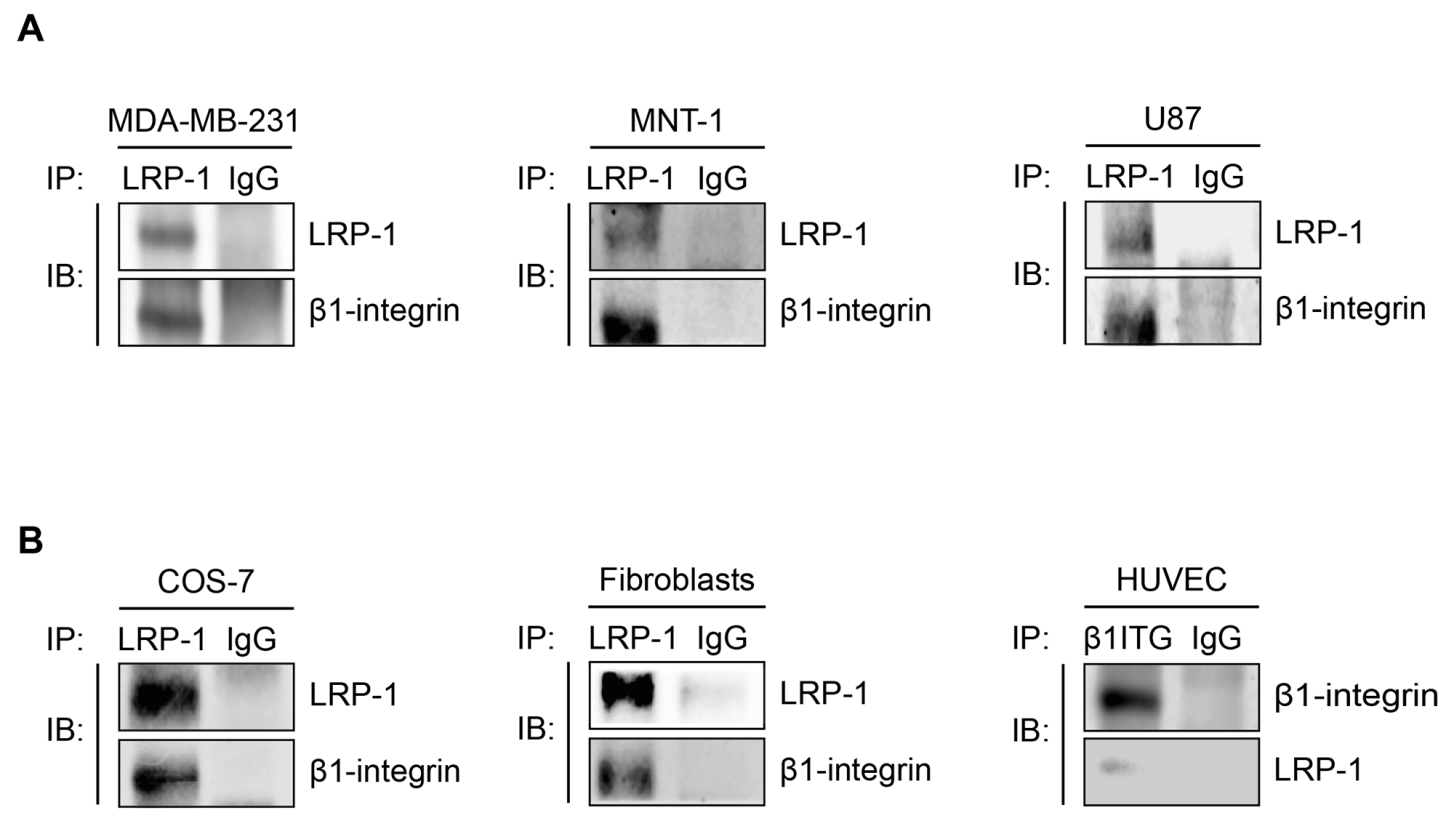
The LRP-1/β1-integrin complex is not only established in thyroid carcinoma Whole-cell extracts were isolated from breast tumor cells (**A**, left panel), melanoma cells (**A,** middle panel), glioblastoma cells (**A,** right panel), COS-7 cells (**B**, left panel), primary dermal fibroblast (**B,** middle panel) or endothelial cells (**B,** right panel). Immunoprecipitation of LRP-1 was conducted and immunocomplexes were immunoblotted using anti-LRP-1 and anti-β1-integrin antibodies as described above. Considering the low level of LRP-1 expression in HUVECs, immunoprecipitations of β1-integrin was favored. Nonspecific IgGs were used as a negative control for each assay.

Interestingly, results from immunoprecipitation assays carried out in various tumor (Figure 3A) and non-tumor cells (Figure 3B) indicated that the LRP-1/β1-integrin complex is not specific of thyroid carcinoma and likewise exists in many cellular environments.

### Endogenous LRP-1 and β1-integrin are colocalized in thyroid carcinoma cells

**Figure 4:**
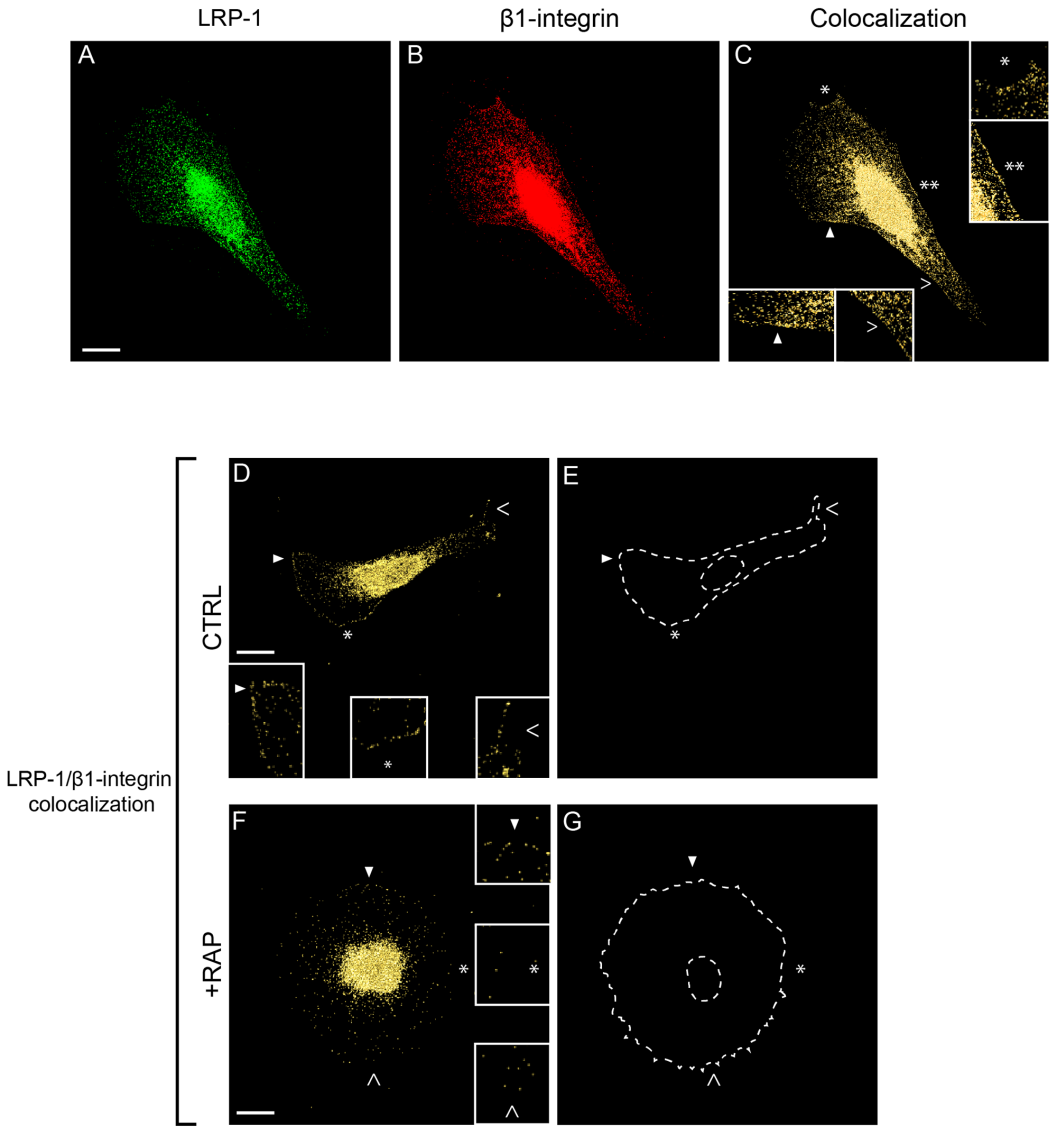
Endogenous β1-integrin colocalizes with LRP-1 in FTC-133 cells FTC-133 cells were seeded onto 1% gelatin-coated coverslips and allowed to grow 24 h in control conditions (**A** to **E**, CTRL) or under 500 nM RAP treatment during 1h (**F-G,** +RAP). Cell labeling was conducted using Alexa Fluor 488 for LRP-1 α-chain **(A)** and Alexa Fluor 568 for β1-integrin **(B)** both validated for immunofluorescence (data not shown) before confocal microscopy analysis. Colocalization between LRP-1 and β1-integrin is represented in yellow in C, D and F panels. Two-fold enlargements (insets) are indicated by empty and full arrowheads or by white stars, highlighting cell membrane regions **(C to G)**. **E** and **G** were used as patterns to represent cell shapes of **D** and **F**, respectively. Images were treated with AMIRA software and correspond to isosurface representations, as indicated in Materials and Methods. Bars: 10 μm.

Having established that a LRP-1/β1-integrin complex occurs markedly at the plasma membrane of thyroid carcinoma cells (Figure 2C), we analyzed the cellular distribution of these proteins using confocal immunofluorescence microscopy (Figure 4). Superposition of LRP-1 (Figure 4A) and β1-integrin (Figure 4B) images revealed that both endogenous proteins partially colocalized in tumor cells (Figure 4C). One could notice that the LRP-1/β1-integrin complexes were remarkably localized at the plasma membrane area, especially at the leading edge and along the lateral sides of the migrating cell (Figure 4C, insets). Regarding the cell shape, control carcinoma cells exhibited a polarized morphology with membrane protrusions typical of migrating cells (Figure 4D and 4E), in contrast with RAP-treated cells which exhibited an expected overspread and highly adherent phenotype (Figure 4F and 4G) [34]. Interestingly, distribution of LRP-1/β1-integrin complexes at the cell membrane was significantly decreased when RAP was used to antagonize LRP-1-mediated endocytosis (Figure 4D to 4G, insets).

### β1-integrin dynamics appears LRP-1 dependent

**Figure 5:**
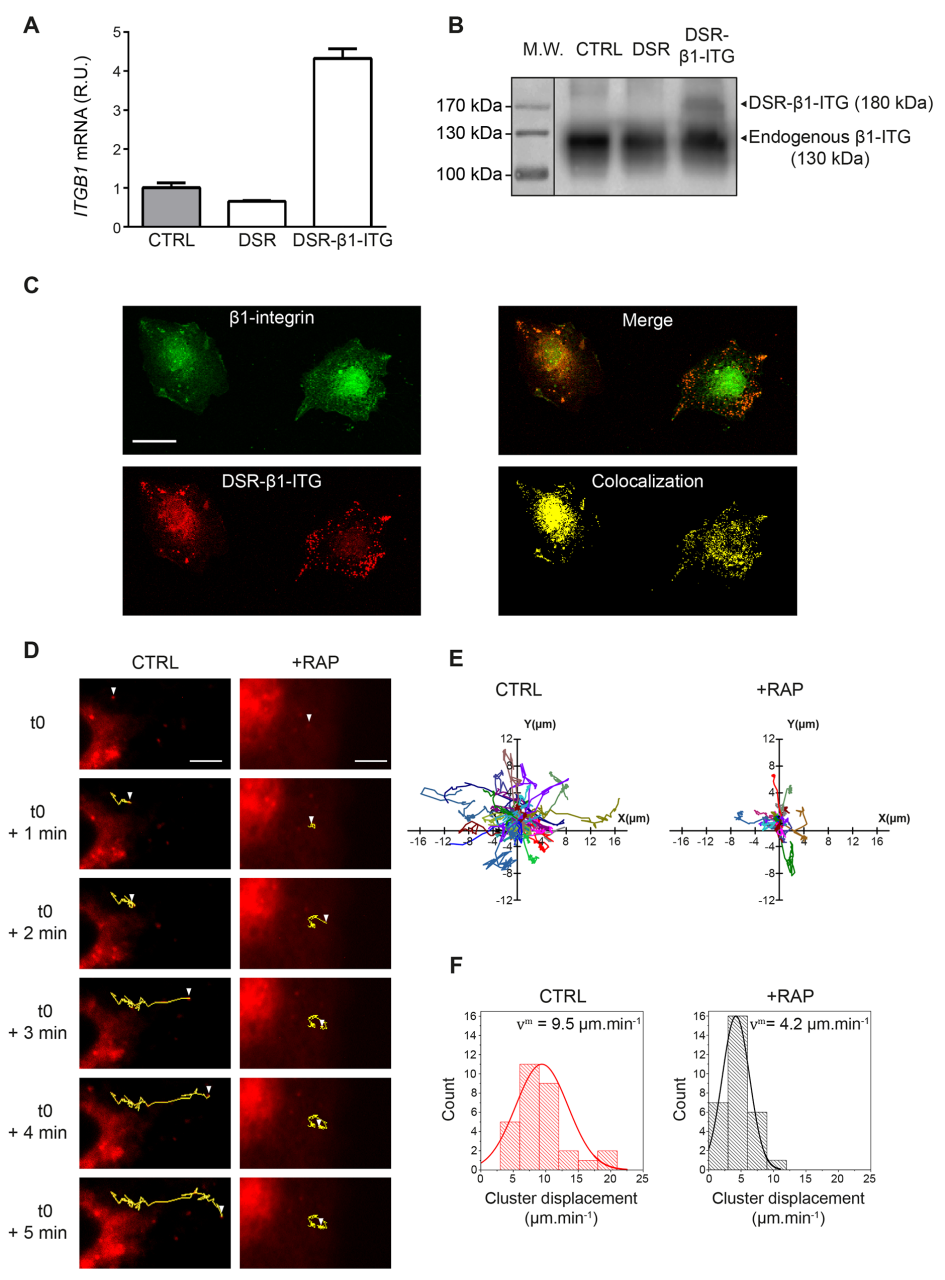
The intracellular trafficking of β1-integrin clusters is under LRP-1 control **(A)** Total RNAs were purified from FTC-133 cells transfected with either a DSR_2_-control plasmid (DSR) or the DsRed_2_-β1-integrin encoding plasmid (DSR-β1-ITG). *ITGB1* mRNA expression was quantified using real-time RT-PCR and expressed as relative units (R.U.). *RPL32* and *RS18* were used for normalization. Non-transfected cells (CTRL) served as a reference set to 1. **(B)** Recombinant DsRed_2_-β1-integrin (DSR-β1-ITG) expression was assessed by SDS-PAGE immunoblotting using anti-β1-integrin (M-106) antibodies. While endogenous β1-integrin migrates around 130 kDa (CTRL and DSR), an expected shift to 180 kDa was observed for the DSR-β1-ITG recombinant product (DSR-β1-ITG). Protein ladder was indicated on the left of the SDS-PAGE. **(C)** FTC-133 cells were seeded onto 1% gelatin-coated coverslides and then allowed to grow for 24 h before being transfected with DsRed_2_-β1-integrin plasmid (DSR-β1-ITG, red). After 24 h, cells were labeled with Alexa fluor 488 for endogenous β1-integrin (green). Merged image and colocalization signal were presented in right panels. Bar: 10 μm. **(D)** FTC-133 cells were seeded onto 1% gelatin coated labtek-I and then transfected with DsRed_2_-β1-integrin plasmid (DSR-β1-ITG). Cells were maintained under control conditions (CTRL) or treated with 500 nM RAP (+RAP) while video microscopy images were taken every 4 s over 5 min. Trajectories for one representative DSR-β1-ITG cluster in both conditions are represented in yellow. White arrowhead represents the position of DSR-β1-ITG cluster from t=0 (i.e. 10 min after RAP addition) to t=5 min. Bars: 5 μm. **(E)** Trajectories of DsRed_2_-β1-integrin clusters obtained under control conditions (CTRL) or 500 nM RAP treatment (+RAP) are represented through *XY* axes (n=28 and n=29, respectively; 4 independent experiments). **(F)** Diagrams represent the repartition of DsRed_2_-β1-integrin clusters displacement speed under control (CTRL) or 500 nM RAP treatment (+RAP) conditions. v^m^ represents the average speed of DSR-β1-ITG clusters in each condition. Overlay curve is the normalized Gaussian curve deduced from our results.

We demonstrated that LRP-1 and β1-integrin closely colocalized in thyroid carcinomas, and we thus investigated whether LRP-1 may contribute to β1-integrin intracellular trafficking. For this purpose, we designed a recombinant DsRed_2_-tagged β1-integrin (DSR-β1-ITG) that was checked for correct mRNA transcription (Figure 5A) as well as protein expression (Figure 5B) in plasmid-transfected thyroid carcinomas. Figure 5C shows that the recombinant DsRed2-tagged β1-integrin exhibited a consistent localization in transfected cells, especially forming clusters at the adhesion sites. Recombinant β1-integrin cluster dynamics was then monitored by video microscopy (Figure 5D to 5F). In control conditions, β1-integrin clusters exhibited large amplitude motions especially along a linear and straightforward displacement (Figure 5D and 5E, left panels). On the contrary, β1-integrin clusters turned around a single point and progressively slowed down to immobilization under RAP-mediated LRP-1 functional inhibition (Figure 5D and 5E, right panels). Accordingly, we found that RAP treatment induced a two-fold decrease in the motion velocity of β1-integrin clusters (Figure 5F).

### β1-integrin addressing to early endosomes occurs in a LRP-1-dependent manner

**Figure 6:**
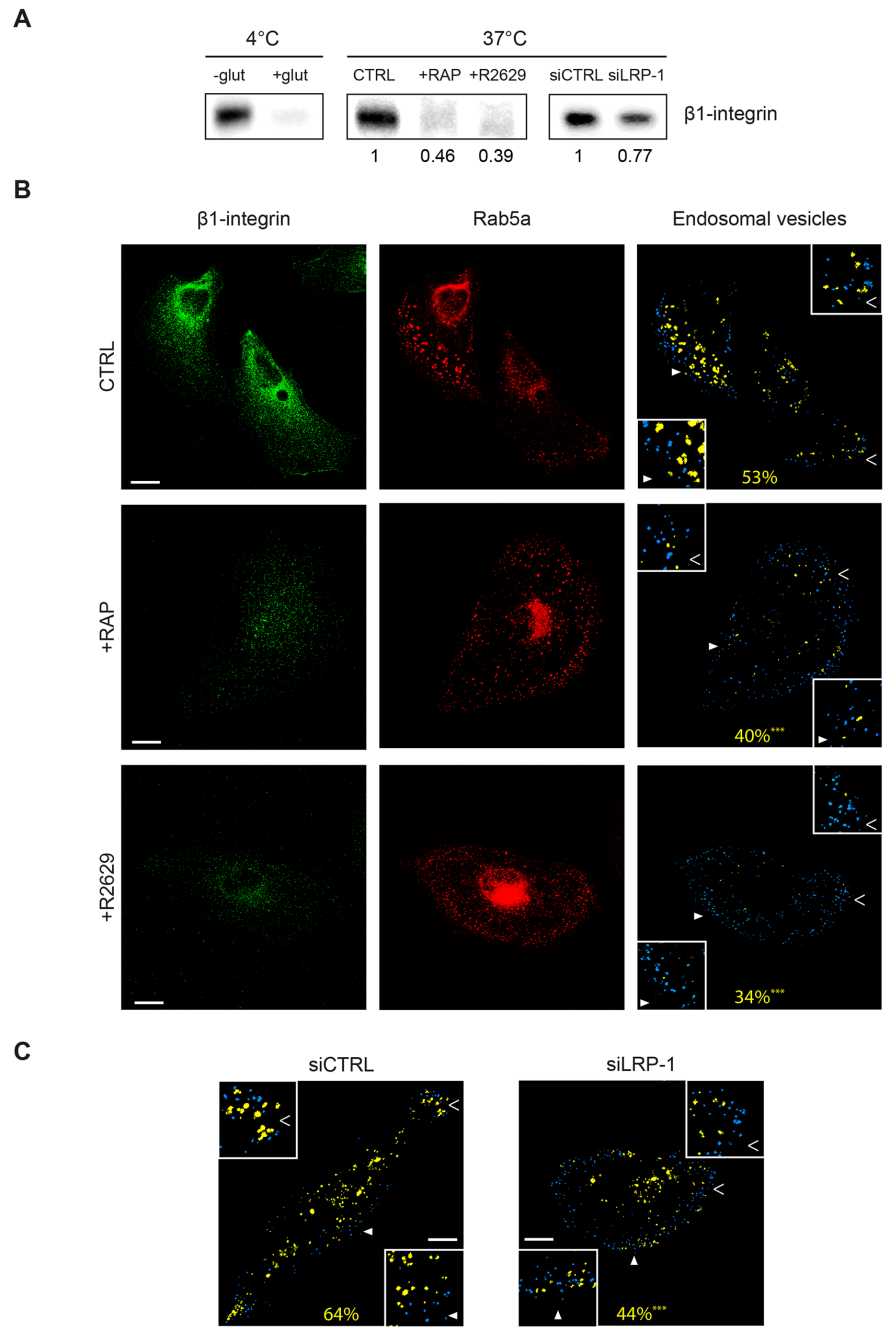
LRP-1 mediates β1-integrin endocytosis in thyroid tumor cells **(A)** FTC-133 cells were plated onto 1% gelatin-coated wells and then allowed to grow for 1 h under non-treated conditions (CTRL), 500 nM RAP (+RAP) or 2.5 μg.mL^-1^ R2629 blocking antibody (+R2629), or either transfected with non-silencing siRNA (siCTRL) or siRNA targeting *LRP1* (siLRP-1). Internalization assay was conducted as described in Materials and Methods section. The amount of internalized β1-integrin was analyzed by immunoblotting using M-106 antibodies (37°C, middle and right frames). Left frame (4°C) serves to control β1-integrin binding to the cell surface (-glut, lane 1) and glutathione efficiency (+ glut, lane 2). Numbers under the immunoblots indicate the average fold induction by comparison with the CTRL (middle panel) or siCTRL (right panel) cells, and were calculated from three distinct experiments. **(B)** FTC-133 cells were cultured onto 1% gelatin-coated coverslips and maintained under control conditions (CTRL), 500 nM RAP treatment (+RAP) or 2.5 μg.mL^-1^ R2629 blocking antibody (+R2629). Then, cells were labeled with Alexa Fluor 488 for β1-integrin (left panel) and infected with endotracker for Rab5a staining (middle panel) before confocal microscopy analysis. On the right panel, endosomal vesicles containing no β1-integrin are represented in blue while β1-integrin-containing-endosomal vesicles are represented in yellow. The average percent of early-endosomes containing β1-integrin is indicated in yellow. Statistical analysis was conducted on endosomal vesicles from 60 to 200 voxels (***, P < 0.001; as compared to CTRL). Insets (full and empty white arrowheads) highlight representative endosomal vesicles in each condition (2-fold enlargement). Bars: 10 μm. **(C)** The same experimental procedure and statistical analysis as that detailed above (in B) were carried out on FTC-133 cells either transfected with non-silencing siRNA (siCTRL) or siRNA targeting *LRP1* (siLRP-1). Bars: 10 μm.

As β1-integrin trafficking occurs under LRP-1 dependence (Figure 5), we sought to determine whether LRP-1 mediates β1-integrin internalization. We investigated β1-integrin uptake by using a previously validated endocytosis assay [37]. This approach requires labeling of cell surface proteins using a non-membrane-permeating sulfo-NHS-LC-biotin at 4°C, then moving to a permissive temperature for endocytosis (37°C). Cell surface protein biotinylation as well as efficiency of biotin stripping with glutathione were controlled (Figure 6A, left panel). In this assay, we determined that 83% of the biotinylated β1-integrin was internalized after 10 min at 37°C in control conditions. Results from Figure 6A (middle panel) indicate that β1-integrin internalization was decreased by about 2-fold and 2.5-fold when LRP-1-mediated endocytosis was inhibited by RAP antagonist or using R2629 blocking antibodies, respectively [38, 39]. Consistently, the uptake of β1-integrin was also partially inhibited under LRP-1 siRNA silencing (Figure 6A, right panel). To investigate this further, Rab5a labeling was performed to examine β1-integrin distribution within early endosome vesicles (Figure 6B). Confocal imaging allowed analysis of β1-integrin-containing early endosomes (Figure 6B, yellow spots in right panels) as well as endosome vesicles that did not contain β1-integrin (Figure 6B, blue spots in right panels). In control conditions, β1-integrin appeared to be highly internalized, particularly from the migrating front (Figure 6B, upper panel). Distribution of β1-integrin into early endosomes was drastically decreased when antagonizing LRP-1-mediated endocytosis with RAP (Figure 6B, middle panel) or using R2629 blocking antibodies (Figure 6B, bottom panel). Quantitative analysis confirmed these observations as the percentage of early endosomes positive for β1-integrin was reduced compared to control cells by 25% and 36% under RAP or R2629 treatment, respectively. In accordance with the foregoing results, β1-integrin internalization was reduced by 31% in LRP-1-silenced cells as compared to control cells transfected with nontargeting siRNA (Figure 6C).

### LRP-1 does not route β1-integrin toward lysosomes and mediates its recycling back to the cell surface

**Figure 7:**
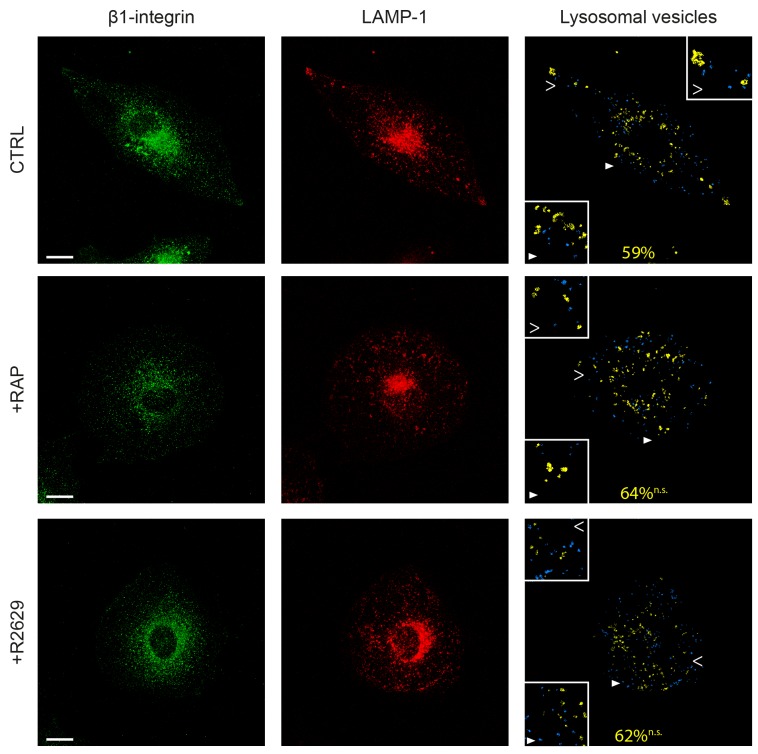
LRP-1 does not address β1-integrin to lysosomes FTC-133 cells were plated onto 1% gelatin coated coverslides and then maintained during 1 h under control conditions (CTRL), 500 nM RAP (+RAP) or 2.5 μg.mL^-1^ R2629 antibody (+R2629) treatment. β1-integrin staining with Alexa Fluor 488 (left panel) and infection with lysotracker for LAMP-1 (middle panel) were carried out before confocal microscopy analysis. On the right panel, lysosomes containing no β1-integrin are represented in blue and β1-integrin-containing lysosomes are represented in yellow. The average percentage of lysosomes positive for β1-integrin is indicated in yellow. Statistical analysis was conducted on lysosomal vesicles from 60 to 200 voxels (n.s., not significant; as compared to CTRL). Cell areas indicated by full and empty white arrowheads highlighting representative lysosomes for each condition are proposed as 2-fold enlargement insets. Bars: 10 μm.

**Figure 8:**
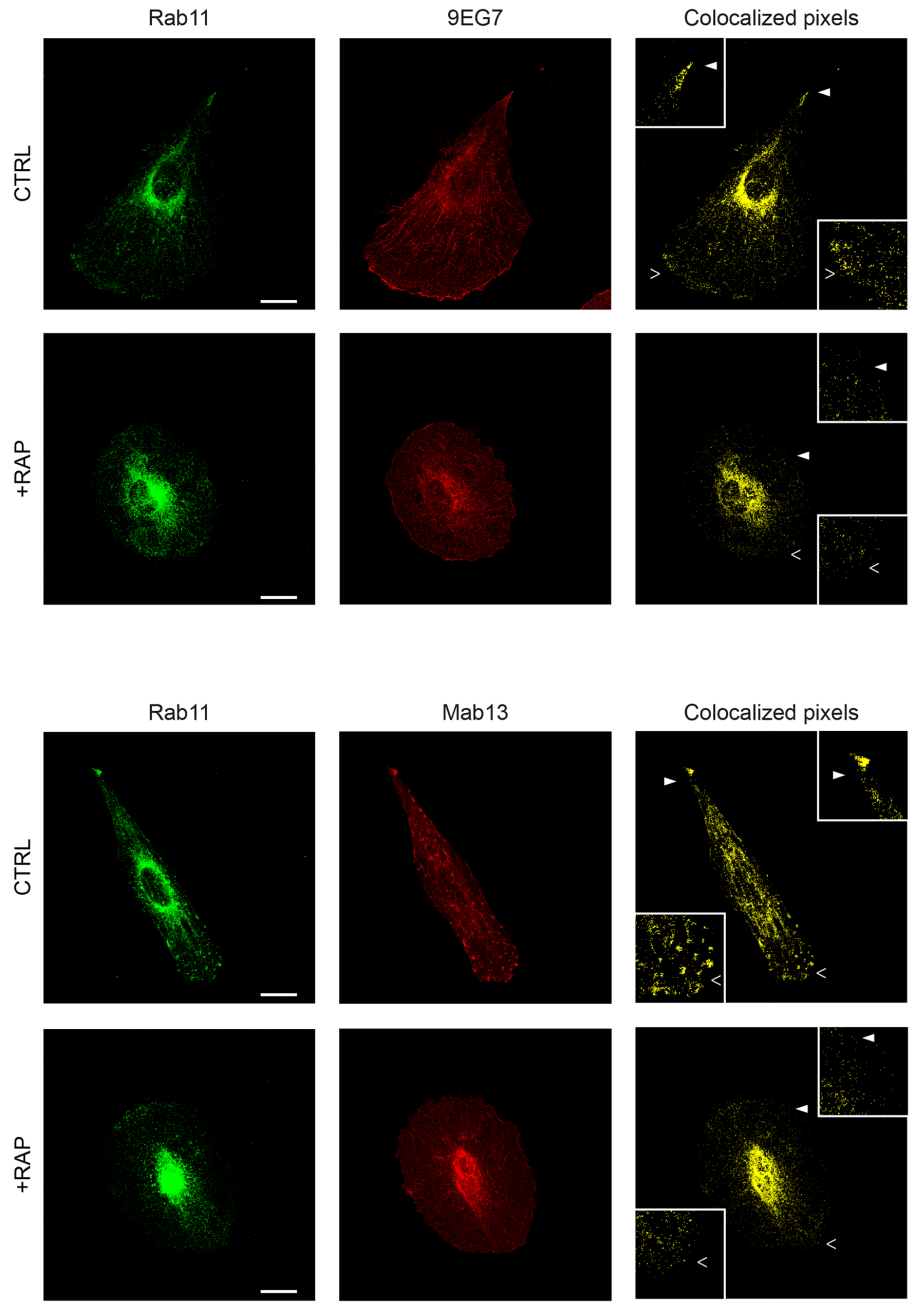
β1-integrin recycling appears to be under LRP-1 dependence in thyroid tumor cells FTC-133 cells were plated onto 1% gelatin coated coverslips, maintained under control conditions (CTRL) or 500 nM RAP treatment (+RAP), and then stained with Alexa Fluor 488 for Rab11 (left panels) and Alexa Fluor 568 for active β1-integrin (9EG7, upper panel) or inactive β1-integrin (Mab13, lower panel) before confocal microscopy analysis. Colocalization between Rab11 and either active or inactive β1-integrin is represented in yellow (colocalized pixels: right panel). 2-fold enlargements (insets) are indicated by full and empty white arrowheads. Bars: 10 μm.

Next we examined whether internalized β1-integrin clusters were delivered to lysosomes in a LRP-dependent manner. For this purpose, cells were labeled using anti-β1-integrin antibodies following infection of a fusion construct of LAMP-1 (lysosomal-associated membrane protein-1) and Tag-RFP (red fluorescent protein) (Figure 7). Interestingly, we observed that 59% of lysosomes contained β1-integrin in control tumor cells (Figure 7, upper panel). Contrary to that has been observed regarding endosome vesicles (Figure 6B), LRP-1 blockade using either RAP or R2629 treatment did not significantly affect the percentage of β1-integrin-containing lysosomes, nor the apparent intracytoplasmic distribution of such vesicles (Figure 7, middle and bottom panels). No other lysosomal features (i.e. number, size) appeared significantly altered by such treatments (data not shown). These results indicate that LRP-1-mediated endocytosis of β1-integrin is not routed toward lysosomes for degradation. To assess the fate of β1-integrin internalized by LRP-1, we carried out immunofluorescence experiments using double labeling with antibodies raised against β1-integrin and Rab11, a marker of recycling endosomes [40]. In control conditions, colocalization of active (9EG7) as well as inactive (Mab13) β1-integrin with Rab11 was mainly observed at the perinuclear area and also at the migration front and the trailing edge of the cell (Figure 8). Such a colocalization signal was drastically decreased under RAP conditions for both active (upper panel) and inactive (lower panel) β1-integrin. These data confirmed that the fraction of β1-integrin internalized by LRP-1 was mainly addressed to the endosomal trafficking pathway for recycling.

## DISCUSSION

In this study, we highlighted that the endocytic receptor LRP-1 establishes tight molecular connections with β1-integrin isoform at the cell surface of thyroid carcinoma cells. In a tumor context, we here provide evidence that the LRP-1/β1-integrin complex constitutes an endocytic complex able to route β1-integrin to early endosomes, then to recycling Rab11-containing vesicles, while avoiding lysosome targeting. We thus identified a new molecular process that regulates the cell surface levels of β1-integrin and that directs it to the endosomal trafficking pathway in thyroid tumor cells.

Our findings provide new mechanistic insights that help to improve the understanding of LRP-1 contribution in cancer cells. Although long debated, it is now well established that LRP-1 may control critical events influencing tumor cell attachment, spreading, migration and invasion [20, 21, 24, 33, 41]. *LRP1* gene silencing in cancer cells was indeed found to accelerate cell attachment, provoke major actin-cytoskeleton rearrangements and induce the accumulation of abundant talin-containing focal adhesion complexes [20, 34]. Focal adhesions are large complexes establishing a link between cell actin cytoskeleton and the ECM. The cytoplasmic side of these complexes consists in an association of many structural, enzymatic and adapter proteins including talin, paxillin and FAK [42]. LRP-1 was shown to decrease talin levels and distribution in cancer cells and is required for paxillin and FAK association within focal adhesions [20, 33]. This sustains the existence of a close functional relationship between LRP-1 and focal adhesions. Orr and collaborators consistently described LRP-1 as a co-receptor for calreticulin which is necessary to stimulate reorganization and disassembly of focal adhesions in endothelial cells in response to the hep I sequence of thrombospondin-1 [29]. It was therefore proposed that the ability of LRP-1 to mediate endocytosis of transmembrane proteins may explain its role in coordinating the adhesion-deadhesion balance of cancer cells. Transmembrane integrins assure a direct link between proteins from the ECM and the cytoplasmic complex. Furthermore, integrins are well characterized for their central role in regulating cell adhesion and migration in both a physiological or tumor context [31, 32, 43]. For all these reasons, we are convinced that LRP-1-mediated internalization and recycling of β1-integrin sheds new light on how LRP-1 contributes to focal adhesion dynamics and disassembly.

Until now, limited data existed in the literature regarding a close functional relationship between LRP-1 and integrins. Our data obtained from thyroid carcinoma cells clearly demonstrate that inhibition of LRP-1-dependent endocytosis by either RAP or RNA interference promotes a similar cell surface accumulation of both β1- and β2-integrin. Considering β2-integrin, it is not particularly surprising because LRP-1 specifically recognizes β2-integrin when associated with αM isoform in macrophages [44, 45]. It was also previously reported that down-regulation of LRP-1 impaired β2-integrin-dependent adhesion of leukocytes to endothelial cells [35]. For β1-integrin, recent work described that accumulation of LRP-1 at the plasma membrane correlated with elevated levels of cell surface β1-integrin in mouse embryonic fibroblasts harboring a knock-in replacement of the distal NPxY motif of LRP-1 [46]. In this work, we have shown that LRP-1 tightly binds with both active and inactive isoforms of β1-integrin (Figure 2) and that both isoforms can be directed *via* LRP-1 to endosomal trafficking (Figure 8). This point deserves to be noted because previous work reported distinct subcellular localization and divergent trafficking routes between active and inactive β1-integrin in breast, prostate and lung cancer cells [36]. Our results demonstrate that both β1-integrin conformations colocalize within Rab11 vesicles, suggesting that both active and inactive conformations undergo common recycling early steps in thyroid carcinomas.

Further, we observed that LRP-1-mediated-endocytosis did not route β1-integrin toward lysosomes (Figure 7). Integrin endocytosis was recently shown to facilitate endosomal FAK signaling which mainly contributes to suppress anoikis and support anchorage-independent growth of cancer cells [47]. From our results, we believe that it is likely that LRP-1 plays a role in such cancer-related processes by directing and maintaining β1-integrin to the endosomal traffic. This is highly coherent with our previous findings stressing the role of LRP-1 in sustaining FAK activation [20, 33]. It was reported that SNX17 (sorting nexin-17) acts as a sorting signal for β1-integrin, directly addressing it toward a Rab11 recycling loop and therefore avoiding lysosomal degradation [48, 49]. Remarkably, SNX17 is also known to bind LRP-1 to facilitate its recycling back to the plasma membrane [50, 51] thus making it a likely molecule involved in the recycling pathway of LRP-1 complexed with β1-integrin.

In thyroid cancer cells, the LRP-1/β1-integrin biomolecular complex was rather observed at the migration front and along the cell body where retraction fibers occur. This is in line with a contribution of LRP-1 in focal adhesion distribution and dynamics [34, 46]. Other studies have described a preferential localization of LRP-1 at the same sites in invasive cancer cells [22, 28]. The LRP-1/β1-integrin complex was also found widely in the perinuclear and the juxtamembrane areas, consistent with its maintenance during internalization and traffic in the cytoplasm. Using a video microscopy approach, we showed that trajectories of β1-integrin clusters were sharply modified when the LRP-1-mediated endocytosis was antagonized, turning out from a straightforward direction to circular motions centered on a single point without any distinctive linear direction (Figure 5). This provides new molecular data regarding integrin traffic.

Using truncated mini-receptors, we demonstrated that the ligand-binding domains II and IV of the LRP-1 α-chain were required for β1-integrin binding (Figure 2). This explains why RAP treatment significantly decreased the LRP-1/β1-integrin complex formation and impaired its distribution and trafficking (Figures 4-6 and 8). This observation seems to contradict recent work reported by Rabiej and collaborators describing the crucial role of the intracellular β-chain of LRP-1 in integrin ligation [46]. To explain this, we suggest that LRP-1 and β1-integrin may not interact in a direct fashion but rather within a biomolecular structure involving other binding partners, which would be highly dependent of the cellular microenvironment. Indeed, this is supported by surface plasmon resonance experiments that failed to reveal direct connections between these two proteins (data not shown). Interestingly, the formation of this complex is not specific of thyroid carcinoma and occurs in many tumor and non-tumor cells (Figure 3), probably depending at least to the amount and localization of LRP-1 at the cell surface.

β1-integrin is able to dimerize with 12 different α partners [52] and among these heterodimers, several are known to promote tumor progression such as α2β1 [53], α3β1 [54], α5β1 [55, 56] or αvβ1 [57]. It is known that β1-integrin association with α5-integrin is recycled through Rab11 vesicles to increase focal adhesion turnover or to influence cell spreading and migration [58]. Despite expression of α2, α3, α5 and αv-integrin in our experimental model, we did not identify a preferential α-subunit associated with β1-integrin during the LRP-1-mediated endocytosis. Furthermore, no α-integrin was found accumulated at the cell-surface under LRP-1 antagonism or silencing (Figure 1). We do not believe that LRP-1 could mediate endocytosis of β1-integrin without an α isoform counterpart. We rather speculate that under LRP-1 inhibition, accumulated β1-integrin at the plasma membrane was redistributed between distinct α isoforms making difficult the detection of a significant change in α/β-integrin levels.

It is worth noting that the amount of β1-integrin at the plasma membrane is only enhanced by about 25% under LRP-1 inhibition (Figure 1). This is in concordance with the 25% to 36% decrease in the number of β1-integrin-containing early endosomes observed under LRP-1 blockade (Figure 6). This means that only one third of the β1-integrin pool is internalized in a LRP-1-dependent manner. This appears relatively low compared to what was reported for other membrane-anchored partners of LRP-1 involved in adhesion, such as CD44 or uPAR [10, 28, 59]. This can be explained by a differential availability of these receptors on the plasma membrane. Of note, LRP-1 was reported to promote β1-integrin maturation and to contribute to increase its amount at the cell surface [60]. Therefore, this may interfere with our observations and may explain why only low accumulation of β1-integrin was detected at the plasma membrane under LRP-1 blockade or silencing.

We believe that the ability of LRP-1 to mediate the uptake and recycling of β1-integrin has consequences on tumor progression beyond the regulation of cell adhesion, migration and invasion. It has been shown that β1-integrin expression dictates radiotherapy resistance in head and neck cancer [61] and drives lung and breast cancer cell resistance to erlotinib and lapatinib, respectively [62, 63]. Particularly, α2β1 integrin was characterized as an important survival pathway in doxorubicin-induced apoptosis [64]. α5β1 heterodimers were further found to support glioma resistance to temozolomide and is being considered as a therapeutic target for high-grade brain tumors [65]. Targeting β1-integrin/mTOR pathway was also suggested as a therapeutic strategy to overcome chemotherapy resistance to docetaxel for the treatment of gliomas [66].

Overall, deciphering the mechanisms of LRP-1-dependent endocytosis is key for increasing our knowledge on how β1-integrin expression, localization and traffic is regulated in tumor cells. By achieving this we have enabled others to consider alternative mechanisms whilst developing alternative drugs targeting β1-integrin.

## MATERIALS AND METHODS

### Antibodies and recombinant proteins

Mouse anti-LRP-1 α-chain (clone 8G1) and β-chain (clone 5A6) monoclonal antibodies were from Calbiochem (San Diego, CA, USA) and rabbit LRP-1-β-chain antibody (clone EPR3724) was from Abcam (Cambridge, UK). Nonreactive rabbit (EPR25A) and mouse (K1814) IgGs used as negative control for immunoprecipitation were from Abcam and Santa Cruz Biotechnology (Heidelberg, Germany), respectively. Blocking LRP-1 polyclonal antibody (R2629) was a generous gift from Dr. D.K. Strickland (Department of Surgery, University of Maryland School of Medicine, Baltimore, MD, USA) [38]. Anti-β1-integrin antibodies were purchased from Abcam (mouse, clone P4G11), Santa Cruz Biotechnology (rabbit, M106) or BD Pharmingen (San Jose, CA, USA) (rat, clone 9EG7 and clone Mab13). Anti-Rab11 (mouse, 47) antibody was purchased from Merck Millipore (Billerica, MA, USA). Alexa Fluor 488, Alexa Fluor 568, Alexa Fluor 647, Hoechst 33342 and FluorSave Reagent were from Thermofisher (Waltham, MA, USA). Anti-hemagglutinin (anti-HA) tag antibody (rabbit, C29F4) was from Cell Signaling (Danvers, MA, USA) and anti-actin (goat, I-19) antibody was purchased from Santa Cruz Biotechnology. FITC-labelled α2-macroglobulin was purchased from BioMac (Leipzig, Germany). Histidine-tagged RAP was purified as previously described [28] and used to antagonize LRP-1-dependent endocytosis as previously reported [28, 33].

### DNA constructs

The HA-tagged mini-receptors derived from LRP-1 were generated as described elsewhere [28]. Briefly, the SPCT (SP: signal peptide; CT: C-terminal) mini-receptor only contains LRP-1 β-chain, whereas DII and DIV molecular constructs respectively encompass the second and fourth ligand binding domains of LRP-1. The mammalian expression vector encoding the chimeric human β1-integrin DsRed_2_ (DSR-β1-ITG) was generated as follows: a cDNA fragment coding for the full-length human β1-integrin was amplified from clone IRATp970E0719D (ImaGenes, Germany) with the primers 5'-GGTAGCTAGCATGAATTTACAACCAATTTTCTGG-3' and 5'-CCGCTCGAGTTTTCCCTCATACTTCGGATTG-3'. After NheI and XhoI digestion, the PCR product was inserted into pcDNA3.1 vector coding for the tandem dimer of DsRed, tdimer2, as previously described [67]. The cDNA construct was then sequenced and the chimeric protein DSR-β1-ITG has a theoretical molecular weight of 143 kDa. Considering post-translational glycosylations, the expected molecular weight on SDS PAGE is about 180 kDa.

### Cell culture and transfection

Human follicular thyroid carcinoma cells (FTC-133 ; ECACC 94060901) were grown in Dulbecco's modified Eagle medium (DMEM)–Ham's F-12 medium (Dutscher, Brumath, France) supplemented with 10% fetal bovine serum (FBS), as previously described [59]. MDA-MB-231 (human breast cancer cells), COS-7 (kidney fibroblasts) and primary dermic fibroblast were cultured in DMEM plus 10% FBS. U87 (human glioblastoma cells) and MNT-1 (human melanoma cells) were grown in Minimum Essential Media (MEM, Dutscher) supplemented with 10% and 20% FBS, respectively. HUVECs (human umbilical vein endothelial cells) were cultured in complete endothelial growth medium (EGM-2, Lonza). FTC-133 cells were transiently transfected with LRP-1-derived mini-receptors or DSR-β1-ITG encoding plasmid using JetPEI reagent (Polyplus Transfection, Illkirch, France) according to manufacturer’s instructions. LRP-1 knockdown was achieved by RNA interference using a previously validated siRNA approach [20]. LRP-1 siRNA as well as non-targeting siRNA used as controls were purchased from Dharmacon (Lafayette, CO, USA) and transiently transfected using Lipofectamine 2000 (Invitrogen, Carlsbad, CA, USA), as reported [28].

### Integrin antibody array

The α/β-integrin-mediated cell adhesion array kit (ECM532, Merck Millipore) was used in order to assess the presence of specific integrin subunits at the cell surface. Experiments were performed as indicated by the manufacturer on untreated, RAP-treated and siRNA-transfected FTC-133 cells.

### RNA isolation and real-time PCR

Total mRNA were extracted using TRIzol reagent (Thermofisher), isolated from other cellular materials by chloroform/isoamyl alcohol (24:1) precipitation before centrifugation (12,000 × *g*, 4°C, 15 min), as described elsewhere [34]. 250 ng total mRNA were reverse-transcribed using VERSO cDNA kit (Thermofisher) according to the manufacturer instructions. Real-time PCR was then performed using an Absolute SYBR Green Rox mix (Thermofisher) and a CFX 96 real time PCR detection system (Bio-Rad, Hercules, CA, USA). The cycle threshold (Ct) values were recorded using Bio-Rad CFX Manager 3.0 software (Bio-Rad) [34]. PCR primers were synthesized by Eurogentec (Liege, Belgium) as follow (5’-3’): for *LRP1*: GCTATCGACGCCCCTAAGAC and CGCCAGCCCTTTGAGATACA; for *ITGB1*: CATCTGCGAGTGTGGTGTCT and GGGGTAATTTGTCCCGACTT; for *RS18*: GCAGAATCCACGCCAGTACAA and GCCAGTGGTCTTGGTGTGCT; for *RPL32*: CATTGGTTATGGAAGCAACAAA and TTCTTGGAGGAAACATTGTGAG.

### Immunofluorescence and internal vesicles labeling

FTC-133 cells were seeded onto 1% gelatin-coated glass slides for 24 h at 37°C and then fixed in phosphate-buffered saline (PBS) containing 4% paraformaldehyde for 15 min at room temperature. After three washes with ice-cold PBS, cells were incubated for 1 h in PBS containing 1% bovine serum albumin and then incubated overnight at 4°C with primary antibodies raised against LRP-1 (clone 8G1), β1-integrin (M-106, clone 9EG7 or Mab13) or Rab11 (clone 47). Then, slides were washed five times with ice-cold PBS and cells were incubated with secondary antibodies conjugated to Alexa Fluor 488 (1/500), Alexa Fluor 568 (1/500) or Alexa Fluor 647 (1/500) during 2 h at room temperature. For endosomes and lysosomes labelling, living cells were incubated overnight at 37°C with CellLight Endosome-RFP or CellLight Lysosome-RFP (Thermofisher) according to the manufacturer instructions, and then fixed in 4% paraformaldehyde for 15 min at room temperature.

### Total protein extraction and western blot analysis

Whole-cell extracts were prepared by scraping cells in ice-cold lysis buffer containing 10 mM Tris-HCl, pH 7.5, 150 mM NaCl, 5 mM EDTA, 1% Triton X-100, supplemented with proteinase inhibitor cocktail (Sigma-Aldrich, Saint-Louis, MO, USA). Samples were vortexed and remaining pellet was separated by centrifugation (10,000 × *g* for 20 min at 4°C) and then discarded. Protein concentration was quantified using the BCA method (Interchim, Montluçon, France). Proteins were separated by sodium dodecyl sulfate-polyacrylamide gel electrophoresis (SDS-PAGE), transferred onto a nitrocellulose membrane (Amersham Biosciences, Little Chalfont, UK), and incubated 16 h at 4°C under gentle agitation with anti-LRP-1 α-chain (1 μg.mL^-1^; 8G1), anti-LRP-1 β-chain (1 μg.mL^-1^, 5A6 or EPR3724), anti-β1-integrin (1 μg.mL^-1^; M-106) or anti-β-actin (0.2 μg.mL^-1^; I-19). Membranes were then incubated for 2 h at room temperature with the corresponding secondary fluorescence-coupled antibody. Tris-buffered saline (TBS)-Tween buffer was used for all washes. Immunoreactive bands were revealed using the Odyssey-FC system (Licor, Lincoln, NE, USA). Immunoblots presented are representative of at least three separate experiments.

### Cell surface protein isolation

FTC-133 cells were washed twice with ice-cold PBS, and then cell surface proteins were biotinylated with PBS containing 0.5 μg.mL^-1^ of EZ-Link sulfo-NHS-LC-biotin (Thermofisher) for 30 min at 4°C. After three washes, cells were incubated with 100 mM glycine for 30 min at 4°C in order to limit nonspecific binding. Cells were washed three times in ice-cold lysis buffer before protein extraction. Cell extracts were pelleted at 10,000 × *g* (20 min, 4°C) before protein quantification. Solubilized biotinylated proteins were then affinity purified using 40 μl of monomeric avidin-agarose beads (GE Healthcare, Chicago, IL, USA) incubated with 120 μg of biotinylated proteins. Incubation was performed overnight at 4°C under gentle orbital agitation (5 rpm), and then followed by five washes in lysis buffer. For immunoblotting experiments, SDS-containing Tris-glycine buffer was added, and samples were heated at 100°C during 5 min, centrifuged (1000 × *g* for 2 min at 4°C), and resolved by SDS-PAGE followed by immunoblotting analysis.

### Immunoprecipitation

Whole-cell extracts or plasma membrane extracts (corresponding to cell surface biotinylated proteins) were subjected to immunoprecipitation using anti-LRP-1 (clone 8G1 or EPR3724), anti-β1-integrin (P4G11) antibodies, or nonspecific IgGs. For immunoprecipitation of biotinylated proteins, a concentration of 10 mM D-biotin in PBS was first used for competitive elution of biotinylated proteins from avidin-agarose beads. Immunoprecipitation was performed by incubating samples with protein G-Sepharose beads (GE Healthcare) for 4 h at 4°C on an orbital agitator. Samples were washed three times in lysis buffer containing no Triton X-100. At last, bead-bound protein complexes were solubilized under non-reducing conditions and then analyzed by immunoblotting as described above.

### Endocytosis and recycling assays

Biotin-based endocytosis assays were adapted from a validated biochemical method [37] and carried out as previously described [28]. FTC-133 cells were allowed to grow in 10% FBS-containing medium in 10 cm dishes until they reached 80% confluency. Cells were then transferred on ice and then washed once with ice-cold PBS. Cell surface proteins were labeled using PBS containing 0.5 mg.mL^-1^ EZ-link cleavable sulfo-NHS-SS-biotin (Thermofisher) for 30 min at 4°C. Unbound biotin was removed with cold PBS before addition of pre-warmed 10% FBS-containing medium. Biotin-labeled surface proteins were allowed to internalize for 10 min at 37°C, and then cells were quickly placed back on ice with cold PBS. After internalization, remaining biotin at the cell surface was removed by washing twice with 50 mM glutathione (Sigma-Aldrich) in an appropriate buffer (10 mM EDTA, 75 mM NaCl, 75 mM NaOH, pH 8.0) for 15 min at 4°C. To determine the total amount of surface biotinylation, one dish was kept on ice after biotin labeling and preserved from glutathione treatment. Cells were washed with ice-cold PBS and then lysed by scraping as described above. 350 μg of biotinylated proteins were immunoprecipitated from the supernatant by adding 40 μL of protein G-sepharose beads (GE Healthcare). Internalized integrins were detected by immunoblotting using anti-β1-integrin antibody (M-106).

### Microscopy

Following plating on gelatin-coated Lab-Tek I chambers, cells were maintained in phenol red-deprived DMEM-F12 (supplemented with low serum growth supplement and 10% FBS) for epifluorescence video microscopy experiments. For bright field video microscopy, cells were observed over a 30 min period using a Zeiss Axiovert 200M equipped with a Coolsnap camera (Roper Scientific, Trenton, NJ, USA). Cells were maintained at 37°C in 5% CO_2_ atmosphere using a temperature-controlled chamber (PECON, Erbach, Germany). Data were acquired using MetaMorph software (Roper Scientific). Timespan between image capture was 4 s. Confocal microscopy images of fixed cells were collected with a Zeiss (Oberkochen, Germany) LSM710 Meta confocal microscope using either a ×63 Plan Apochromat objective (oil immersion, 1.40 NA, DIC) at a 132 nm.pixel^-1^ resolution, leading to a slight *XY* oversampling. Optical slices were spaced by 1 μm so as to limit *Z* oversampling. Fluorescence filters were chosen and tested for each combination of plasmid construction and fluorescent dye so as to abolish spectral overlap. Zen software program was used to acquire images.

### Image processing

Isosurface representations were realized using the AMIRA software program (v6.2; Visage Imaging, Berlin, Germany) as previously detailed in [28]. Acquired confocal stacks for vesicle (endosome/lysosome) labeling were analyzed with a custom made Matlab script in order to determine the number of integrin-containing vesicles and to estimate the corresponding integrin amount from pixel intensity. Briefly, the script integrates data from a pair of stacks (vesicle labeling and integrin labeling). Images were first thresholded and then the fluorescence channels that correspond to vesicle immunostaining (i.e. Rab5a from endosome or LAMP-1 for lysosome) were analyzed to determine the pixels that are in close contact to each other (26-connected neighborhood) and therefore define the volume of each vesicle considered separately. Any vesicles <7 voxels were considered as artefact and removed from data analysis accordingly. The contour of remaining vesicles was then smoothed and compared to the channels corresponding to integrin labeling. The enabled, number/volume/shape of each vesicle to be measured, as well as the amount of labeled integrins contained within these vesicles. The script was automated in order to analyze wide collections of stacks therefore increasing the number of measurements. For video microscopy experiments, β1-integrin DsRed_2_ (DSR-β1-ITG) cluster trajectories were manually determined using the Point Picker ImageJ plugin.

### Data analysis

All results presented raised from at least three independent experiments. Two sample bilateral *t*-test and ANOVA were used to compare assays (Prism software; GraphPad).

## Abbreviations

ADAMTS: a disintegrin and metalloproteinase with thrombospondin motifs
DSR-β1-ITG: DsRed_2_-tagged β1-integrin
ECM: extracellular matrix
ERK: extracellular signal-regulated kinases
FAK: focal adhesion kinase
FBS: fetal bovine serum
JNK: c-jun N-terminal kinase
LAMP-1: lysosomal-associated membrane protein-1
LRP-1: low-density lipoprotein receptor-relapted protein-1
MMP: matrix metalloproteinase
PAI-1: plasminogen activator inhibitor
RAP: receptor-associated protein
RFP: red fluorescent protein
SNX17: sorting nexin-17
uPA: urokinase plaminogen activator
uPAR: urokinase plaminogen activator receptor
